# 

**Published:** 1880-04

**Authors:** 


ESTABLSIHED IN 1855.

VOLUME XVIII. APRIL, 1880. NUMBER 1

### ATLANTA MEDICAL AND SURGICAL JOURNAL.

S.G.O. INDEXED

*MONTHLY: THREE DOLLARS A YEAR, IN ADVANCE.*

S.G.O. INDEXED

EDITOR:

J. G. WESTMORELAND, M.D.,

*Professor of Materia Medica in Atlanta Medical College.*

SURGEON GENL'S OFFICE LIBRARY. 82409

H. H. DICKSON, PUBLISHER.

*PAX ET SCIENTIA, SED VERITAS SINE TIMORE.*

ATLANTA, GEORGIA:

*H. H. DICKSON, BOOK AND JOB PRINTER AND BOOKBINDER.*

1880.

